# Associations between *XRCC1* Gene Polymorphisms and Coronary Artery Disease: A Meta-Analysis

**DOI:** 10.1371/journal.pone.0166961

**Published:** 2016-11-21

**Authors:** Wen-Qi Ma, Xi-Qiong Han, Xin Wang, Ying Wang, Yi Zhu, Nai-Feng Liu

**Affiliations:** Department of Cardiology, Zhongda Hospital, School of Medicine, Southeast University, 87 Dingjiaqiao, Nanjing, 210009, P.R. China; Sudbury Regional Hospital, CANADA

## Abstract

Genetic variations that influence DNA repair efficiency may contribute to coronary artery disease (CAD) susceptibility. Previous studies have investigated whether there was evidence of an association between polymorphisms at the X-ray repair cross complementing 1 (*XRCC1*) gene and susceptibility to CAD, but findings have been inconclusive. We identified eligible studies through a comprehensive literature search to determine whether an association exists between *XRCC1* gene polymorphisms and CAD susceptibility. Findings were assessed using the odds ratio (OR) and corresponding 95% confidence interval (CI), which were calculated using a fixed- or random-effects model, based on the heterogeneity of the studies. Ten eligible studies were finally included in this meta-analysis. Our pooled analysis found that *XRCC1* polymorphisms were significantly associated with CAD susceptibility under recessive (Arg194Trp: OR = 1.47, 95% CI = 1.13–1.93; Arg399Gln: OR = 1.45, 95% CI = 1.12–1.89), homozygous (Arg194Trp: OR = 1.37, 95% CI = 1.03–1.81; Arg399Gln: OR = 1.56, 95% CI = 1.19–2.05), and allele (Arg399Gln: OR = 1.18, 95% CI = 1.06–1.32) genetic models. Following subgroup analysis by ethnicity, in Asian populations, we found evidence of associations between the XRCC1 Arg194Trp polymorphism and CAD under recessive and homozygous genetic models, and between the XRCC1 Arg399Gln polymorphism and CAD under recessive, homozygous, and allele genetic models. Subgroup analysis stratified by control source revealed associations between the Arg194Trp and Arg399Gln polymorphisms and susceptibility to CAD under recessive and homozygous modes of inheritance, respectively. In addition, subgroup analysis stratified by sample size found that findings of the Arg194Trp polymorphism in large sample sizes were comparable to those found using pooled eligible studies. Based on our meta-analysis, we concluded that the *XRCC1* gene polymorphisms, Arg194Trp and Arg399Gln, are associated with CAD susceptibility, specifically in Asian populations. However, additional, comprehensive and well-designed studies are warranted to confirm these findings.

## Introduction

Coronary artery disease (CAD), which is recognized as a major public health problem, has high mortality and morbidity worldwide [1]. The main underlying cause of CAD is atherosclerosis. As a chronic, progressive, and multifactorial disease, a variety of complex mechanisms affects the initiation and progression of atherosclerosis and plaque vulnerability [2, 3]. One possible mechanism for the progression of atherosclerosis is via DNA damage, which is caused by the generation of oxidative stress, the accumulation of reactive oxygen species (ROS), the metabolism of toxic byproducts, and ionizing radiation [4–6]. Genome integrity is probably influenced by endogenous and exogenous DNA damage, and sustained deleterious effects that block both replication and transcription of DNA may lead to mutations and chromosomal aberrations [7, 8]. However, several DNA damage response pathways, such as base excision repair (BER), nucleotide excision repair (NER), and single strand break repair (SSBR), fix such DNA damage [9]. One of the most important components for the efficient repair of SSBR and BER is X-ray repair cross complementing protein 1 (XRCC1) [10–12]. It has a role as a scaffold coordinating other proteins in the DNA repair complex. The *XRCC1* gene is located on chromosome 19q13.2–13.3, it consists of 17 exons and encodes a protein of 633 amino acids [13].

Two *XRCC1* gene polymorphisms, Arg194Trp (rs1799782) and Arg399Gln (rs25487), which are located at codon 194 of exon 6 and at codon 399 of exon 10 respectively, are reported to affect the expression of *XRCC1* and subsequently influence the DNA repair capacity [14, 15]. Furthermore, previous studies have found that individuals with DNA repair deficiencies show an increased sensitivity to CAD [16–18]. In addition, several epidemiological studies have revealed that these two variations in the *XRCC1* gene influence DNA repair and are associated with an increased susceptibility to CAD [19–21]. For example, Bazo et al. [22] demonstrated that individuals possessing the T allele in Arg194Trp or the A allele in Arg399Gln have an increased likelihood of CAD compared to that found in individuals with the wild-type alleles of either polymorphisms. However, other studies did not consistently find evidence of an association between polymorphisms in the *XRCC1* gene and the likelihood of developing CAD [18, 23].

Whether a correlation exists between variants in the DNA repair gene *XRCC1* and susceptibility to CAD remains controversial and inconclusive. Furthermore, no relevant meta-analysis or genome-wide association studies (GWAS) have been published on this subject. Consequently, to derive a more comprehensive estimation of the role of *XRCC1* gene polymorphisms in CAD susceptibility, we identified all eligible studies and performed this meta-analysis.

## Methods and Materials

### Literature search strategy

The literature search was performed by two authors (Ma and Zhu). Comprehensive electronic databases including PubMed, EMBASE, Web of Science, ScienceDirect, Cochrane Library, and Ovid were systematically searched for eligible studies up to July 31, 2016. Our search strategy was based on finding keywords or topic-based classifications in the title and abstract of published studies. Keywords and terms used were (“XRCC1” OR “X-ray repair cross-complementing group 1” OR “Arg194Trp” OR “rs1799782” OR “Arg399Gln” OR “rs25487”) and (“genetic polymorphism” OR “allele” OR “genotype” OR “variant”) and (“coronary artery disease” OR “myocardial infarction” OR “angina” OR “atherosclerosis”). References in eligible studies were also examined as a secondary source to identify eligible studies. Manual searching of relevant journals and screening the reference lists were performed by one author (Liu).

### Inclusion and exclusion criteria

Studies included in our meta-analysis were selected according to the following inclusion criteria: (1) the study examined the correlation between the *XRCC1* Arg194Trp and Arg399Gln polymorphisms and CAD; (2) patients with CAD were documented using angiographic evidence of at least 50% stenosis of one major coronary artery, myocardial infarction, angina, or a history of coronary artery bypass surgery; (3) the total number of cases and controls, distribution of genotypes, or other relevant data could be extracted from the references; and (4) the publication language was in English. Studies were excluded if they met any of the following criteria: (1) publications were abstracts, letters to the editor, animal studies, or reviews; (2) the study presented data that overlapped with a previous publication; and (3) the study presented either uninformative data or did not provide genotype frequencies.

### Data extraction

The data from eligible studies were extracted by two independent authors (Ma and Han). Disagreements regarding study selection were resolved by a third author (Liu). Critical data that could not be derived from the references were obtained by directly contacting the authors. We collected the following information: author, year of publication, ethnicity, country, the number of cases and controls, baseline characteristics of the patients, genotyping methods, genotype frequencies in cases and controls, the origin of the controls, and measurements of Hardy-Weinberg equilibrium (HWE) from controls.

### Quality assessment

The quality of eligible studies was assessed by one author (Han), according to the Newcastle–Ottawa scale (NOS) for genetic association studies [24]. NOS quality scores ranged between 0 and 9 stars. Studies with a score ≥ 7 were considered high quality, while studies with a score ≤ 5 were deemed low quality.

### Statistical analysis

All statistical analyses were performed using Review Manager v5.2 (The Cochrane Collaboration, Oxford, UK) and Stata 12.0 (Stata Corporation, College Station, Texas, USA). Pooled odds ratios (ORs) and 95% confidence intervals (CIs) were calculated to evaluate the robustness of the association between *XRCC1* gene polymorphisms and CAD susceptibility. Tests under dominant (Arg194Trp: CT + TT vs. CC; Arg399Gln: GA + AA vs. GG), recessive (Arg194Trp: TT vs. CT + CC; Arg399Gln: AA vs. GA + GG), homozygous (Arg194Trp: TT vs. CC; Arg399Gln: AA vs. GG), heterozygous (Arg194Trp: CT vs. CC; Arg399Gln: GA vs. GG), and allele (Arg194Trp: T vs. C; Arg399Gln: A vs. G) genetic models were performed and ORs were determined. The Cochrane Q-test and index (I^2^) were used to examine heterogeneity among studies with a *P* > 0.10 in the Q-test indicating no heterogeneity. I^2^ was used to estimate the total variation across studies where an I^2^ < 25% was considered to have a low level of heterogeneity, an I^2^ between 25% and 50% indicated moderate heterogeneity, and an I^2^ > 50% represented a high-level of heterogeneity. A fixed- or random-effects model was performed depending on the observed heterogeneity of the studies. Subgroup analyses were performed to identify potential sources of heterogeneity. Sensitivity analysis was performed to assess the stability of individual studies, and publication bias was determined with funnel plots and Egger’s linear regression test.

## Results

### Selection and characteristics of studies

**Fig 1:**
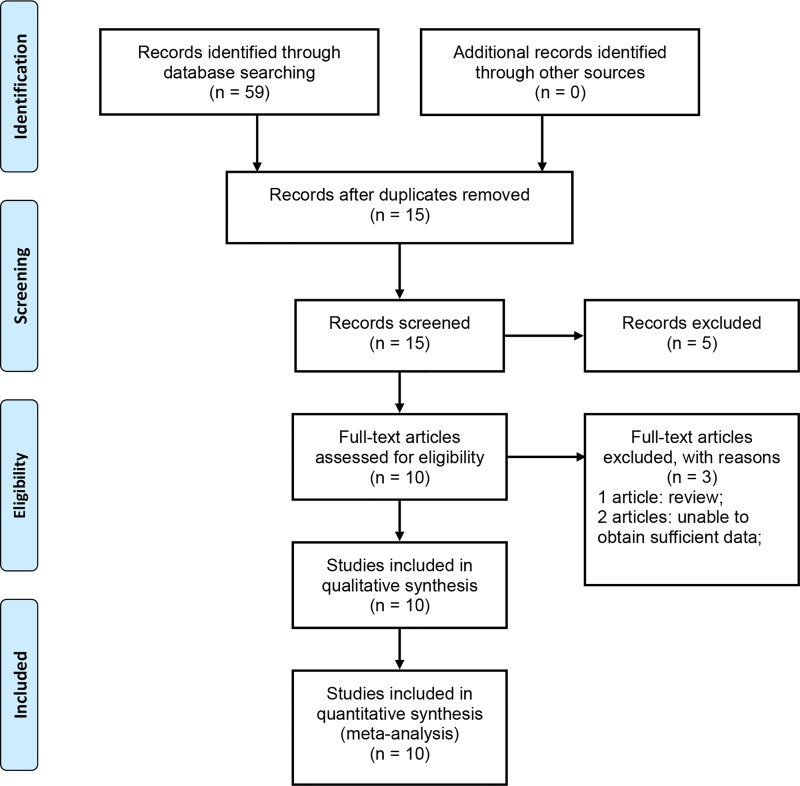
Flow chart to identify eligible studies for meta-analysis.

Fifty-nine articles were selected following an initial search, of which 44 articles were excluded because of duplicate records. Ten articles remained from the original literature search after screening the titles and abstracts. Of these studies, one article was excluded because it was a review and two other articles were excluded because of insufficient data. Finally, seven articles that contained ten studies were retained [18–23, 25]. The flow diagram of our literature selection process is shown in Fig 1.

Information of the selected studies is shown in Table 1. For the Arg194Trp polymorphism, 1,490 cases and 1,458 controls were available from four studies, whereas for the Arg399Gln polymorphism, 1,862 cases and 1,568 controls were available from six studies. The countries of eligible studies included Brazil, Pakistan, China, Turkey, India, and Iran. Two studies used Caucasian samples and six studies used Asian samples. The controls of six studies were hospital-based, whereas those of the other four studies were population-based. Control cohorts from all studies were in HWE (*P* > 0.05).

**Table 1:** Characteristics of eligible studies included in the meta-analysis.

First Author/Year	Country	Ethnicity	Disease Type	Clinical Criteria for Patients	Control Source	Genotyping Methods	Sample Size	No. of Cases	No. of Controls	*P*_*HWE*_ Y/N	NOS
**Arg194Trp**								**CC/CT/TT**	**CC/CT/TT**		
Bazo 2011[22]	Brazil	Mixed	CAD	stenosis >50% in at least one coronary artery	HB	PCR	46/39	40/6/0	28/10/1	Y	6
Yu 2014[20]	China	Asian	CAD	stenosis >50% in at least one major coronary artery or major branches	HB	PCR-LDR	1142/1106	517/486/139	483/531/92	Y	6
Pahlavanneshn 2016[19]	Iran	Asian	CAD	stenosis >50% in at least one coronary artery	HB	PCR-RFLP	203/207	155/44/4	185/19/3	Y	6
Hameed 2016[25]	Pakistan	Asian	AMI, stable and unstable angina	stenosis >50% in at least one coronary artery	PB	PCR-RFLP	99/106	94/3/2	99/4/3	Y	7
**Arg399Gln**								**AA/AG/GG**	**AA/AG/GG**		
Guven 2007^[^^23^^]^	Turkey	Caucasian	CAD	stenosis >50% in at least one coronary artery	PB	PCR-RFLP	147/48	50/76/21	12/33/3	Y	6
Bazo 2011[22]	Brazil	mixed	CAD	stenosis >50% in at least one coronary artery	HB	PCR	117/52	25/76/16	20/28/4	Y	6
Narne 2013[21]	India	Asian	CAD, MI	Diabetic patients with stenosis >50% in at least one coronary artery	HB	PCR	160/121	52/84/24	58/49/14	Y	6
Gokkusu 2013[18]	Turkey	Caucasian	CAD, ACS	stenosis >50% in one major coronary artery	PB	PCR	197/135	169/27/1	119/16/0	Y	7
Yu 2014[20]	China	Asian	CAD	stenosis >50% in one major coronary artery or major branches	HB	PCR-LDR	1142/1106	625/437/80	627/419/60	Y	6
Hameed 2016[25]	Pakistan	Asian	AMI, stable and unstable angina	stenosis >50% in at least one coronary artery	PB	PCR-RFLP	99/106	40/28/31	53/30/23	Y	7

Abbreviations: CAD, coronary artery disease; ACS, acute coronary artery syndrome; AMI, acute myocardial infarction; MI, myocardial infarction; PCR, polymerase chain reaction; PCR-RFLP, polymerase chain reaction-restriction fragment length polymorphism; PCR-LDR, polymerase chain reaction-ligase detection reaction; HWE, Hardy–Weinberg equilibrium; PB, population-based; HB, hospital-based; NOS, Newcastle–Ottawa quality scale.

### Meta-analysis results

Associations between *XRCC1* gene polymorphisms and CAD susceptibility are shown in Table 2. Subgroup analyses were performed based on the source of the controls (hospital-based and population-based controls), ethnicity (Asian and non-Asian populations), and sample size (studies with ≥ 300 subjects were categorized as “large” and studies with < 300 subjects were categorized as “small”) to detect sources of heterogeneity (Table 2).

**Table 2:** Summary of meta-analysis of the association between *XRCC1* gene polymorphisms and coronary artery disease.

Genetic Model	Overall and Subgroup		Arg194Trp				Arg399Gln		
		N	OR (95% CI)	I^2^ (%)	*P*	N	OR (95% CI)	I^2^ (%)	*P*
	**Overall**	4	1.02 (0.51–2.07)	81%	0.001	6	1.32 (0.97–1.79)	56%	0.040
	**PB**	1	0.75 (0.23–2.45)	N/A	N/A	3	1.11 (0.67–1.77)	37%	0.210
	**HB**	3	1.08 (0.46–2.52)	87%	0.000	3	1.56 (0.94–2.59)	76%	0.020
**Dominant model**	**Large sample size**	2	0.99 (0.56–4.10)	92%	0.000	2	1.09 (0.93–1.28)	0%	0.710
	**Small sample size**	2	1.28 (0.23–1.17)	0%	0.410	4	1.47 (0.91–2.38)	59%	0.060
	**Asian populations**	3	1.18 (0.59–2.82)	84%	0.002	3	1.37 (0.94–1.98)	63%	0.070
	**non-Asian populations**	1	0.38 (0.13–1.15)	N/A	N/A	3	1.23 (0.61–2.47)	66%	0.050
	**Overall**	4	1.47 (1.13–1.93)	0%	0.630	6	1.45 (1.12–1.89)	0%	0.910
	**PB**	1	0.71 (0.12–4.33)	N/A	N/A	3	2.09 (1.05–3.16)	0%	0.840
	**HB**	3	1.50 (1.14–1.97)	0%	0.580	3	1.36 (1.01–1.83)	0%	0.830
**Recessive model**	**Large sample size**	2	1.34 (1.16–2.00)	0%	0.890	2	1.32 (0.94–1.86)	0%	0.780
	**Small sample size**	2	0.55 (0.12–2.62)	0%	0.620	4	1.66 (1.10–2.49)	0%	0.850
	**Asian populations**	3	1.32 (1.14–1.96)	0%	0.710	3	1.38 (1.04–1.82)	0%	0.830
	**non-Asian populations**	1	0.28 (0.01–6.97)	N/A	N/A	3	2.16 (0.95–4.90)	0%	0.950
	**Overall**	4	1.03 (0.47–2.27)	82%	0.000	6	1.23 (0.89–1.70)	56%	0.040
	**PB**	1	1.13 (0.17–3.62)	N/A	N/A	3	0.96 (0.59–1.57)	34%	0.220
	**HB**	3	1.08 (0.43–2.70)	88%	0.000	3	1.51 (0.90–2.54)	74%	0.020
**Heterozygous model**	**Large sample size**	2	1.49 (0.47–4.69)	93%	0.000	2	1.05 (0.89–1.25)	0%	0.720
	**Small sample size**	2	1.27 (0.21–1.29)	0%	0.510	4	1.33(0.76–2.34)	66%	0.030
	**Asian populations**	3	1.30 (0.51–3.31)	86%	0.000	3	1.28 (0.87–1.89)	59%	0.090
	**non-Asian populations**	1	0.42 (0.14–1.92)	N/A	N/A	3	1.13 (0.54–2.39)	70%	0.040
	**Overall**	4	1.37 (1.03–1.81)	0%	0.630	6	1.56 (1.19–2.05)	0%	0.780
	**PB**	1	0.70 (0.11–4.30)	N/A	N/A	3	1.78 (0.98–3.23)	0%	0.993
	**HB**	3	1.39 (1.05–1.85)	0%	0.550	3	1.51 (1.11–2.05)	10%	0.330
**Homozygous model**	**Large sample size**	2	1.42 (1.07–1.89)	0%	0.880	2	1.35 (0.95–1.91)	0%	0.780
	**Small sample size**	2	0.53 (0.11–2.49)	0%	0.560	4	1.97 (1.26–3.06)	0%	0.870
	**Asian populations**	3	1.39 (1.05–1.85)	0%	0.750	3	1.49 (1.11–1.98)	0%	0.590
	**non-Asian populations**	1	0.23 (0.01–5.97)	N/A	N/A	3	2.37 (0.98–5.74)	0%	0.790
	**Overall**	4	1.04 (0.58–1.86)	77%	0.004	6	1.18 (1.06–1.32)	24%	0.250
	**PB**	1	1.15 (0.28–1.98)	N/A	N/A	3	1.25 (0.95–1.65)	0%	0.410
	**HB**	3	1.11 (0.55–2.23)	84%	0.002	3	1.17 (1.04–1.32)	56%	0.100
**Allele model**	**Large sample size**	2	1.04 (0.70–3.18)	89%	0.003	2	1.11 (0.97–1.26)	0%	0.670
	**Small sample size**	2	1.24 (0.27–1.10)	0%	0.370	4	1.40 (1.14–1.72)	0%	0.420
	**Asian populations**	3	1.28 (0.17–2.33)	79%	0.009	3	1.17 (1.04–1.32)	53%	0.120
	**non-Asian populations**	1	0.38 (0.14–1.08)	N/A	N/A	3	1.26 (0.94–1.69)	7%	0.340

Abbreviations: XRCC1, X-ray repair cross complementing protein 1; N, number of studies; OR, odds ratio; CI, confidence interval; *P*, *P* value for association; PB, Population-based; HB, Hospital-based; N/A, not available.

**Fig 2:**
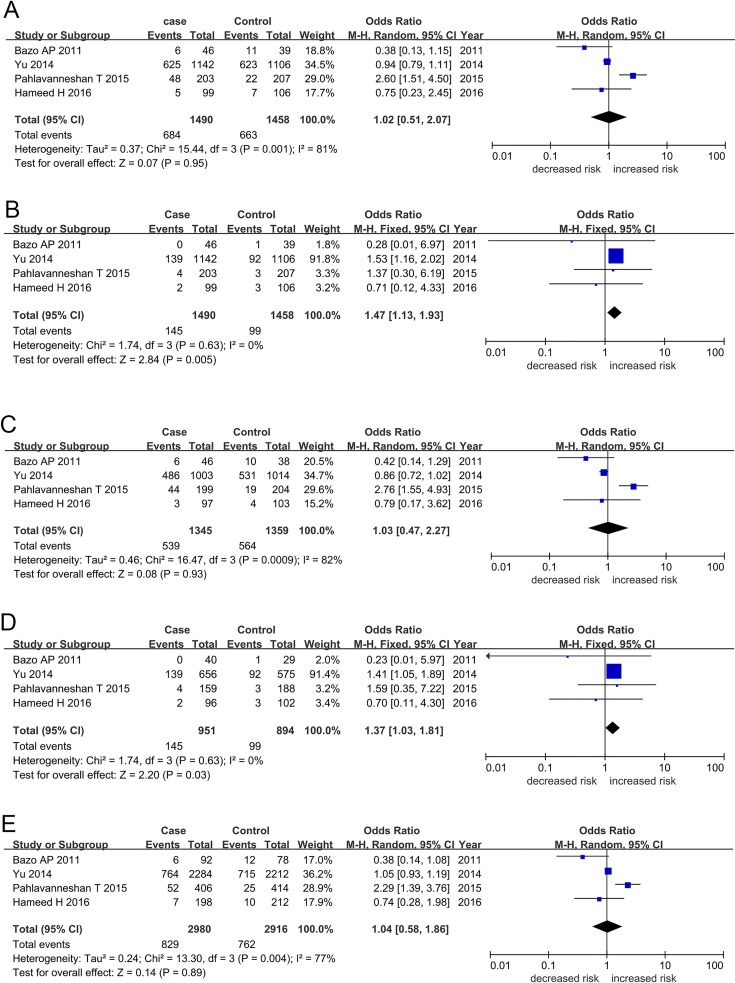
**Forest plots for *XRCC1* Arg194Trp polymorphism and coronary artery disease susceptibility in the genetic models:** (A) Dominant (CT + TT vs. CC), (B) Recessive (TT vs. CT + CC), (C) Heterozygous (TC vs. CC), (D) Homozygous (TT vs. CC), and (E) Allele (T allele vs. C allele). Abbreviations: XRCC1, X-ray repair cross complementing protein 1; CI, confidence interval.

**Fig 3:**
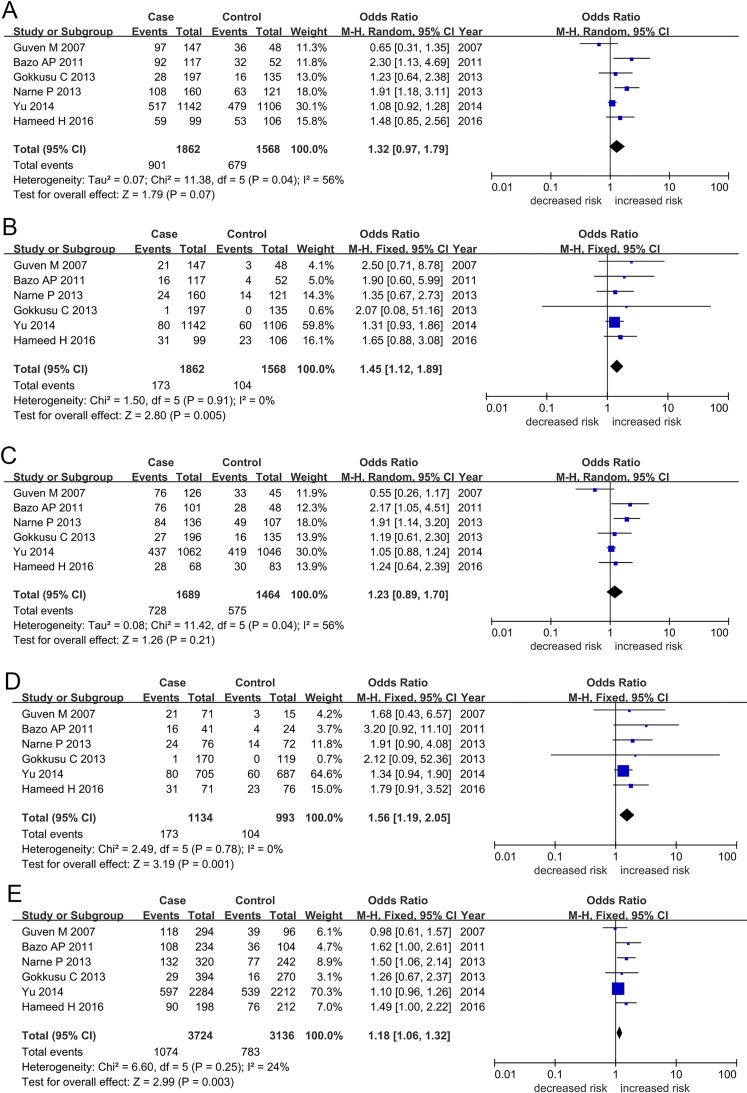
**Forest plots for *XRCC1* Arg399Gln polymorphism and coronary artery disease susceptibility in the genetic models:** (A) Dominant (GA + AA vs. GG), (B) Recessive (AA vs. GA + GG), (C) Heterozygous (GA vs. GG), (D) Homozygous (AA vs. GG), and (E) Allele (A allele vs. G allele). Abbreviations: XRCC1, X-ray repair cross complementing protein 1; CI, confidence interval.

For the *XRCC1* Arg194Trp polymorphism, we found an increased likelihood of CAD susceptibility in recessive (TT vs. CT + CC: OR = 1.47, 95% CI = 1.13–1.93) and homozygous (TT vs. CC: OR = 1.37, 95% CI = 1.03–1.81) genetic models when cohorts from all eligible studies were pooled (Table 2; Fig 2). We found no evidence of an association between CAD and dominant (CT + TT vs. CC: OR = 1.02, 95% CI = 0.51–2.07), heterozygous (CT vs. CC: OR = 1.03, 95% CI = 0.47–2.27), or allele (T vs. C: OR = 1.04, 95% CI = 0.58–1.86) genetic models. Similarly, pooled analysis found evidence of association between the *XRCC1* Arg399Gln polymorphism and CAD susceptibility in recessive (AA vs. GA + GG: OR = 1.45, 95% CI = 1.12–1.89), homozygous (AA vs. GG: OR = 1.56, 95% CI = 1.19–2.05), and allele (A vs. G: OR = 1.18, 95% CI = 1.06–1.32) genetic models, but no evidence of association was identified under dominant and heterozygous models of inheritance (Table 2; Fig 3). We found no between-study heterogeneity under recessive (Arg194Trp: TT vs. CT + CC, I^2^ = 0%, *P*_*Heterogeneity*_ = 0.630; Arg399Gln: AA vs. GA + GG, I^2^ = 0%, *P*_*Heterogeneity*_ = 0.910), homozygous (Arg194Trp: TT vs. CC, I^2^ = 0%, *P*_*Heterogeneity*_ = 0.630; Arg399Gln: AA vs. GG, I^2^ = 0%, *P*_*Heterogeneity*_ = 0.780), and allele (Arg399Gln: AA vs. GG, I^2^ = 24%, *P*_*Heterogeneity*_ = 0.250) genetic models.

For subgroup analyses based on ethnicity, we found evidence of association between the Arg194Trp polymorphism and CAD susceptibility in Asian populations under recessive and homozygous genetic models, and support for an association for the Arg399Gln polymorphism under recessive, homozygous, and allele models of inheritance. Subgroup analyses stratified by control source found evidence of an association for both the Arg194Trp and Arg399Gln polymorphisms with increased likelihood of CAD in hospital-based studies under recessive and homozygous genetic models and a significant association between the Arg399Gln polymorphism and CAD susceptibility in population-based studies under recessive genetic model. In addition, we found in subgroup analyses stratified by sample size that findings related to the Arg194Trp polymorphism from studies with large sample sizes were similar to those found using pooled eligible studies (shown in Table 2).

### Sensitivity analysis

**Fig 4:**
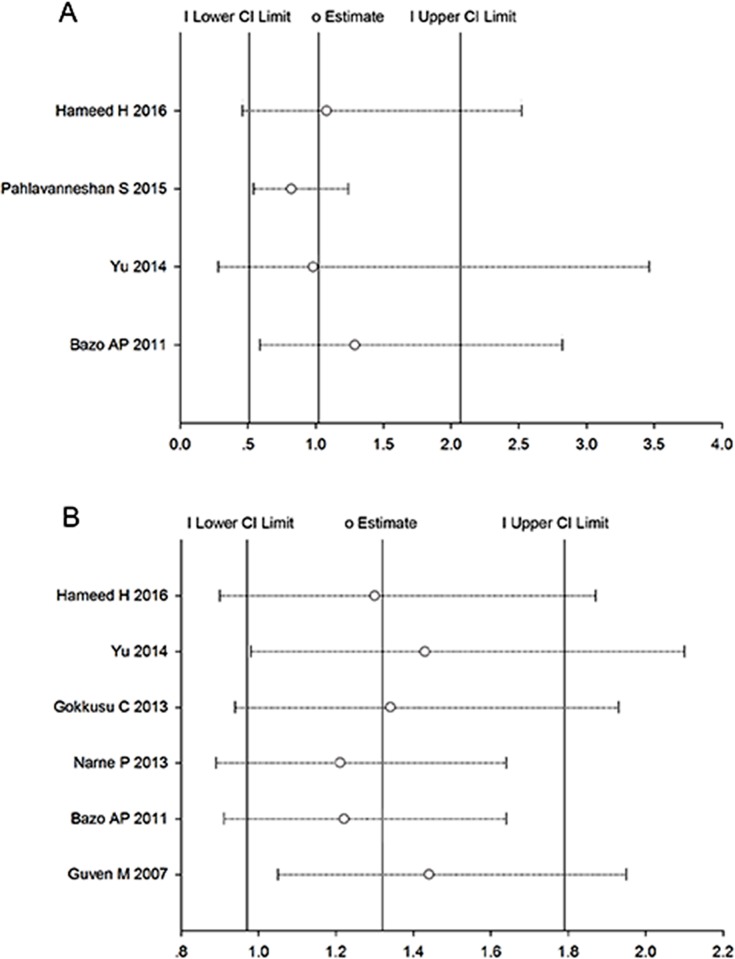
Sensitivity analysis of the correlation between *XRCC1* gene polymorphisms and susceptibility to coronary artery disease. (A) Sensitivity analysis for the Arg194Trp polymorphism. (B) Sensitivity analysis for the Arg399Gln polymorphism. Abbreviations: XRCC1, X-ray repair cross complementing protein 1; CI, confidence interval.

The influence of each study on pooled ORs and 95% CIs were evaluated by excluding each study one at a time. As shown in Fig 4, we found no individual study affected the pooled OR.

### Publication bias

**Fig 5:**
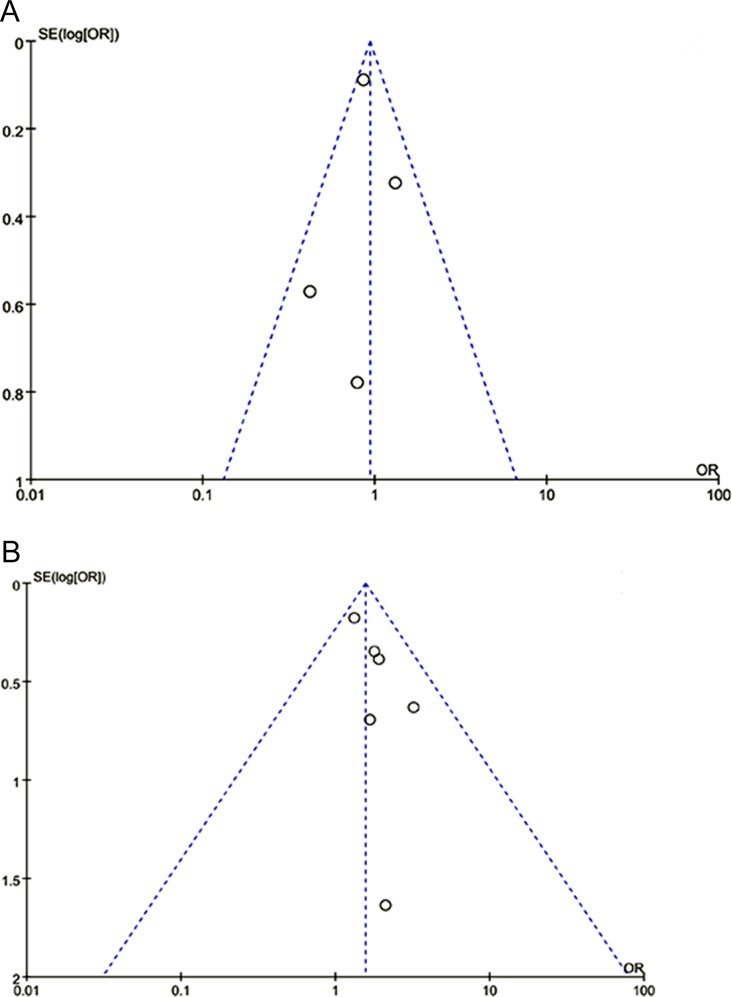
Funnel plots for studies investigating the effect of *XRCC1* polymorphisms on coronary artery disease susceptibility. (A) Funnel plot for publication bias in the Arg194Trp polymorphism. (B) Funnel plot for publication bias in the Arg399Gln polymorphism. Abbreviations: XRCC1, X-ray repair cross complementing protein 1; OR, odds ratio.

Funnel plots (Fig 5) and Egger’s linear regression test found no evidence of publication bias for either of the *XRCC1* polymorphisms in Arg194Trp (TT + CT vs. CC, *P*_*Egger's*_ = 0.173) or Arg399Gln (GA + AA vs. GG, *P*_*Egger's*_ = 0.549).

## Discussion

We analyzed ten studies in our meta-analysis and found evidence for associations between the Arg194Trp and Arg399Gln polymorphisms at the *XRCC1* gene and an increased likelihood of CAD. Furthermore, subgroup analyses based on ethnicity found that carriers of the Arg194Trp T allele or the Arg399Gln A allele were at increased susceptibility to CAD in Asian populations under several genetic models. However, we found no evidence of an association in non-Asian populations, a finding that may indicate differences in genetic diversity between different ethnic groups. Stratification based on control source found that both the Arg194Trp and Arg399Gln polymorphisms increased the likelihood of CAD in hospital-based studies under recessive and homozygous genetic models, and a significant association was observed between the Arg399Gln polymorphism and CAD susceptibility in population-based studies under recessive genetic model. Possible explanations for these discrepant findings may be the limitation of the small sample sizes, different risk factors among the hospital- and population-based controls, and the possibility of selection bias. In addition, it is worth noting that the hospital-based controls are more 'case-like' than the population-based controls for many variables, which may underestimate the effects and can’t represent the general population. NOS was applied for quality assessment and all the studies included were of moderate or high quality, which improved the reliability of the conclusion. Otherwise, studies of low quality might have increased the heterogeneity and produced misleading results. Furthermore, we performed sensitivity analysis and confirmed the robustness of our findings.

Although atherosclerosis is a complex process and its molecular mechanisms are not fully understood, research findings support the hypothesis that DNA damage caused by the production of excessive ROS has an important role in atherogenesis [26, 27], and that such ROS-induced DNA damage is coordinated to some extent by the XRCC1 pathway. Furthermore, polymorphisms in the *XRCC1* gene affect DNA repair efficiency and therefore, may influence individual susceptibility to both atherosclerosis and CAD. Consequently, our findings on the joint effects of these polymorphisms in the DNA repair gene *XRCC1*, on CAD susceptibility supports this rationale and is biologically plausible.

Ahmadi et al. [28] reported increased expression of the *XRCC1* gene in patients with CAD when compared with normal people, and Martinet et al. [29] found a greater number of DNA strand breaks and an overexpression of the *XRCC1* gene in atherosclerotic plaques. Such upregulation may be in response to elevated DNA damage because of atherosclerotic progression. These findings are consistent with the results of our meta-analysis that found *XRCC1* gene polymorphisms may be susceptibility factors and outcome predictors to CAD. However, these findings are in contrast to those of Gokkusu et al. [18] and Guven et al. [23] that did not find evidence of a direct link between *XRCC1* gene polymorphisms and CAD susceptibility. Possible explanations accounting for these inconsistencies between results may be because of genetic heterogeneity across different ethnicities, different baseline characteristics of study subjects, different sample sizes, or cultural differences in external factors such as nutritional status, diet, or daily exercise. On the other hand, the effect of a single polymorphism in the *XRCC1* gene on CAD susceptibility may be very small and is vulnerable to various factors. In recent decades, the dynamic equilibrium between DNA damage and repair in atherosclerosis has been highlighted. However, it remains to be determined whether reducing the rate of DNA damage and improving the capacity of DNA repair, such as overexpression of XRCC1 protein or gene replacement therapy in CAD patients with DNA repair deficiencies, can delay atherosclerotic progression, or open new avenues for therapeutic intervention [30, 31].

Previous meta-analyses have evaluated genetic polymorphisms in the *XRCC1* gene and likelihood for different cancers including thyroid cancer [32], breast cancer [33], glioma [34], and leukemia [35], but to our knowledge, we have performed the first meta-analysis to examine the role of the *XRCC1* gene in CAD susceptibility. Although our study provides a better understanding of genetic variations in *XRCC1* and susceptibility to CAD, it has limitations. First, the limited number of original studies that were examined and the unpublished data that were not available may affect the reliability of our conclusions and potentially limit further analyses. Second, because original individual data could not be extracted from each study and our results were based on unadjusted estimates, the introduction of heterogeneity in our study is unavoidable and may affect our results. Third, because of anticipated interactions between hereditary and environmental factors, genetic variation at a single locus will be insufficient to completely elucidate gene-disease associations. Finally, the publication language of our studies was limited to English and therefore, there is the potential for publication bias, although we found no evidence of such from funnel plots and Egger's test in our meta-analysis.

## Conclusions

The current meta-analysis supports associations between the polymorphisms Arg194Trp and Arg399Gln, in the DNA repair gene *XRCC1*, and increased susceptibility to CAD, specifically in Asian populations. Although subgroup analyses to investigate potential sources of heterogeneity did not find evidence for bias, future large-scale well-designed studies to bolster the robustness of our findings and reliability of our conclusions are warranted.

## Supporting Information

S1 File meta-analysis-on-genetic-association-studies-form. (DOCX)

S2 File PRISMA Checklist. (DOC)

S3 File The full details of databases searching terms. (DOC)

S4 File Full-text articles excluded with reasons. (DOC)

S5 File Eight excluded records. (ZIP)
