# Supplementary material for: Associations between XRCC1 Gene Polymorphisms and Coronary Artery Disease: A Meta-Analysis
Source: PLoS One. 2016 Nov 21;11(11):e0166961. doi: 10.1371/journal.pone.0166961 (PMC5117741; doi:10.1371/journal.pone.0166961)
Supplement: S1 File — (DOCX) [file pone.0166961.s001.docx]

**Meta-analysis on Genetic Association Studies Checklist | PLOS ONE**

|  | Item | Section name and paragraph number within manuscript |
| --- | --- | --- |
|  | **Introduction** |  |
| 1 | Provide a detailed justification for the polymorphism studied; if a single polymorphism was analyzed, give details as to why others were not included in the meta-analysis. | Introduction: paragraph 1-2; |
| 2 | Provide a detailed justification for the population(s) and clinical condition studied. | Introduction: paragraph 1-2 |
|  | **Methods** |  |
| 3 | Provide full details of the search strategy employed; outline the full electronic search strategy –specific combination of keywords and any limits applied- for at least one database. Specify whether synonyms of polymorphisms/genes (e.g. SNP number) were searched. | Methods and materials:  ( Literature search strategy) |
| 4 | Report full details on the inclusion and exclusion criteria applied for selecting studies.  *Please list the excluded articles and the reasons for exclusion of each article in a supplementary file.* | Methods and materials:  (Inclusion and exclusion criteria) |
| 5 | Provide details on how the quality of the studies included in the analyses was assessed. | Methods and materials:  (Quality assessment) |
| 6 | Describe steps taken to contact study authors to identify additional studies and to request missing data. | Methods and materials:  ( Literature search strategy) |
| 7 | Describe how environmental effects were adjusted for, if this adjustment was not conducted, outline the reasons for this. | In our meta-analysis, the included studies did not describe and analysis environment effects, consequently, we did not conduct it. |
| 8 | Describe the methods of handling heterogeneity/between-study variance. | Methods and materials:  (Statistics analysis). |
| 9 | Describe how the Hardy-Weinberg equilibrium and linkage disequilibrium were assessed. | Methods and materials:  (Statistics analysis). |
| 10 | Describe and justify the choice of model for the analyses (per-allele vs per-genotype vs genetic model-free, random effects vs fixed effects). | Methods and materials:  (Statistics analysis).  . |
| 11 | Describe whether a sensitivity analysis has been completed. | Methods and materials: (Statistics analysis).  Results: (Sensitivity analysis and publication bias) |
| 12 | Describe whether an assessment of the effects of population stratification has been conducted. | Methods and materials:  (Statistics analysis). |
| 13 | Describe whether study-specific results have been assessed and if so the reasons for this (e.g. forest plot). | Results: (Meta-analysis results and Forest plot, Fig 2-3) |
|  | **Results** |  |
| 14 | Include flow diagram for the studies included in the meta-analysis as the first figure for the manuscript | Results: Selection and characteristics of studies ( flow diagram: Fig 1) |
| 15 | Report details on allele/genotype prevalence. | Results: Selection and characteristics of studies (Table 1) |
| 16 | Report the effect size estimates and p values for each analysis. | Results: Selection and characteristics of studies (Table 1) |
|  | **Discussion** |  |
| 17 | Discuss the limitations of the meta-analysis, including genotyping errors/bias and publication bias. | In the part of Discussion: Several limitations in our meta-analysis should be addressed as well. First, the limited number of original studies that were examined and the unpublished data that were not available may affect the reliability of our conclusions and potentially limit further analyses. Second, because original individual data could not be extracted from each study and our results were based on unadjusted estimates, the introduction of heterogeneity in our study is unavoidable and may affect our results. Third, because of anticipated interactions between hereditary and environmental factors, genetic variation at a single locus will be insufficient to completely elucidate gene-disease associations. Finally, the publication language of our studies was limited to English and therefore, there is the potential for publication bias, although we found no evidence of such from funnel plots and Egger's test in our meta-analysis. |
| 18 | If the meta-analysis identifies an association within a subgroup of the population studied but not another, discuss the implications of these results, and if applicable the possibility of subgroup-specific publication bias. | This part can be found in the beginning of Discussion section. |
| 19 | Discuss the suitability of the sample size employed to the research question and the power of the study. | This part can be found in the beginning of Discussion section. |
