# Supplementary material for: Associations between XRCC1 Gene Polymorphisms and Coronary Artery Disease: A Meta-Analysis
Source: PLoS One. 2016 Nov 21;11(11):e0166961. doi: 10.1371/journal.pone.0166961 (PMC5117741; doi:10.1371/journal.pone.0166961)
Supplement: S3 File — (DOC) [file pone.0166961.s003.doc]

**Databases searching terms**

(update to 31, July, 2016)

**1. Pubmed Database (n=10)**

((((((((((((Artery Disease, Coronary[Title/Abstract]) OR Artery Diseases, Coronary [Title/Abstract]) OR Coronary Artery Diseases[Title/Abstract]) OR Disease, Coronary Artery[Title/Abstract]) OR Diseases, Coronary Artery[Title/Abstract]) OR Coronary Arteriosclerosis[Title/Abstract]) OR Arterioscleroses, Coronary[Title/Abstract]) OR Acute Coronary Syndrome[Title/Abstract]) OR angina[Title/Abstract]) OR myocardial infarction[Title/Abstract]) OR coronary heart disease[Title/Abstract])) AND (((((XRCC1[Title/Abstract]) OR Arg194Trp[Title/Abstract]) OR Arg399Gln[Title/Abstract]) OR rs25487[Title/Abstract]) OR rs1799782[Title/Abstract])

**2. EMBASE Database (n=15)**

#14. 'coronary artery disease'/exp OR 'coronary artery disease' AND ([embase]/lim OR [medline]/lim) OR ('coronary artery disease':ab AND ([embase]/lim OR [medline]/lim)) OR ('coronary heart disease':ab AND ([embase]/lim OR [medline]/lim)) OR (myocardial AND infarction:ab AND ([embase]/lim OR [medline]/lim)) OR ('angina':ab AND ([embase]/lim OR [medline]/lim)) AND ('xrcc1 protein'/exp AND ([embase]/lim OR [medline]/lim) OR (xrcc1: ab AND ([embase]/lim OR [medline]/lim)) OR (arg194trp: ab AND ([embase]/lim OR [medline]/lim)) OR ('rs1799782': ab AND ([embase]/lim OR [medline]/lim)) OR ('arg399gln': ab AND ([embase]/lim OR [medline]/lim)) OR ('rs25487':ab AND ([embase]/lim OR [medline]/lim)))

#13. 'xrcc1 protein'/exp AND ([embase]/lim OR [medline]/lim) OR (xrcc1: ab AND ([embase]/lim OR [medline]/lim)) OR (arg194trp: ab AND ([embase]/lim OR [medline]/lim)) OR ('rs1799782': ab AND ([embase]/lim OR [medline]/lim)) OR ('arg399gln': ab AND ([embase]/lim OR [medline]/lim)) OR ('rs25487': ab

AND ([embase]/lim OR [medline]/lim))

#12. 'rs25487': ab AND ([embase]/lim OR [medline]/lim)

#11. 'arg399gln': ab AND ([embase]/lim OR [medline]/lim)

#10. 'rs1799782': ab AND ([embase]/lim OR [medline]/lim)

#9. arg194trp: ab AND ([embase]/lim OR [medline]/lim)

#8. xrcc1: ab AND ([embase]/lim OR [medline]/lim)

#7. 'xrcc1 protein'/exp AND ([embase]/lim OR [medline]/lim)

#6. 'coronary artery disease'/exp OR 'coronary artery disease' AND ([embase]/lim OR [medline]/lim) OR ('coronary artery disease':ab AND ([embase]/lim OR [medline]/lim)) OR ('coronary heart disease':ab AND ([embase]/lim OR [medline]/lim)) OR (myocardial AND infarction:ab AND

([embase]/lim OR [medline]/lim)) OR ('angina':ab AND ([embase]/lim OR [medline]/lim))

#5. 'angina':ab AND ([embase]/lim OR [medline]/lim)

#4. myocardial AND infarction:ab AND ([embase]/lim OR [medline]/lim)

#3. 'coronary heart disease':ab AND ([embase]/lim OR [medline]/lim)

#2. 'coronary artery disease':ab AND ([embase]/lim OR [medline]/lim)

#1. 'coronary artery disease'/exp OR 'coronary artery disease' AND ([embase]/lim OR [medline]/lim)

**3. ScienceDirect Database (n=6)**

TITLE-ABSTR-KEY ("XRCC1" OR "rs1799782" OR "Arg194Trp" OR "rs25487" OR "Arg399Gln" OR " X-ray repair cross-complementing group ") and TITLE-ABSTR-KEY ("coronary artery disease" OR "coronary heart disease" OR "angina" OR "myocardial infarction" OR "CAD" OR "atherosclerosis" OR "Coronary Arteriosclerosis" OR "acute coronary syndrome").

**4. OVID Database (n=15)**

1 (XRCC1 or Arg194Trp or X-ray repair cross-complementing group 1 or Arg399Gln or rs25487 or rs1799782). ab.

2 limit 1 to abstracts

3 (coronary artery disease or coronary heart disease or myocardial infarction or angina or CAD or atherosclerosis or Coronary Arteriosclerosis or acute coronary syndrome). ab.

4 limit 3 to abstracts

5 2 and 4

**5. Web of Science Database (n=13)**

TS= ((((((((((((((coronary artery disease) OR coronary heart disease) OR CAD) OR angina) OR atherosclerosis) OR myocardial infarction) OR Artery Disease, Coronary) OR Artery Diseases, Coronary) OR Coronary Arteriosclerosis) OR Atherosclerosis, Coronary) OR Atheroscleroses, Coronary) OR acute coronary syndrome)) AND (((((((Arg194Trp) OR Arg399Gln) OR rs25487) OR -77T>C) OR) OR X-ray repair cross-complementing group 1) OR XRCC1))

**6. Cochrane library (n=0)**

"XRCC1" or "X-ray repair cross-complementing group 1" or "Arg194Trp" or "rs1799782" or "Arg399Gln" or "rs25487": ti, ab, kw and "coronary artery disease" or "myocardial infarction" or "angina" or "atherosclerosis": ti, ab, kw
