# Supplementary material for: Associations between XRCC1 Gene Polymorphisms and Coronary Artery Disease: A Meta-Analysis
Source: PLoS One. 2016 Nov 21;11(11):e0166961. doi: 10.1371/journal.pone.0166961 (PMC5117741; doi:10.1371/journal.pone.0166961)
Supplement: S4 File — (DOC) [file pone.0166961.s004.doc]

Dear Prof,

Thank you for your reminding. First, when searching the electronic databases, the following articles repeated in these databases, therefore we exclude the duplication articles(No.1-12):

1. Pahlavanneshan S, Ahmadi A, Boroumand M, Sadeghian S, Behmanesh M. **Association of -77T>C and Arg194trp polymorphisms of XRCC1 with risk of coronary artery diseases in Iranian population**. Iran J Basic Med Sci. 2016;19(2):194-200. PubMed PMID: 27081465; PubMed Central PMCID: PMC4818368.

2. Ahmadi A, Behmanesh M, Boroumand MA, Tavallaei M. **Up-regulation of MSH2, XRCC1 and ATM genes in patients with type 2 diabetes and coronary artery disease.** Diabetes Res Clin Pract. 2015;109(3):500-6. doi: 10.1016/j.diabres.2015.05.049. PubMed PMID: 26088318.

3. Skarpengland T, Laugsand LE, Janszky I, Luna L, Halvorsen B, Platou CG, et al. **Genetic variants in the DNA repair gene NEIL3 and the risk of myocardial infarction in a nested case-control study. The HUNT Study**. DNA Repair (Amst). 2015;28:21-7. doi: 10.1016/j.dnarep.2015.01.013. PubMed PMID: 25703835.

4. Yu X, Liu J, Zhu H, Xia Y, Gao L, Dong Y, et al. **Synergistic association of DNA repair relevant gene polymorphisms with the risk of coronary artery disease in northeastern Han Chinese**. Thrombosis research. 2014;133(2):229-34. doi: 10.1016/j.thromres.2013.11.017. PubMed PMID: 24315498.

5. Gokkusu C, Cakmakoglu B, Dasdemir S, Tulubas F, Elitok A, Tamer S, et al. **Association between genetic variants of DNA repair genes and coronary artery disease**. Genet Test Mol Biomarkers. 2013;17(4):307-13. doi: 10.1089/gtmb.2012.0383. PubMed PMID: 23368530.

6. Narne P, Ponnaluri KC, Singh S, Siraj M, Ishaq M. **Arg399Gln Polymorphism of X-Ray Repair Cross-Complementing Group 1 Gene Is Associated with Angiographically Documented Coronary Artery Disease in South Indian Type 2 Diabetic Patients. Genetic Testing and Molecular Biomarkers**. 2013;17(3):236-41. doi: 10.1089/gtmb.2012.0330.

7. Bazo AP, Salvadori DJ, Salvadori RA, Sodre LP, da Silva GN, de Camargo EA, et al. **DNA repair gene polymorphism is associated with the genetic basis of atherosclerotic coronary artery disease**. Cardiovasc Pathol. 2011;20(1):e9-15. doi: 10.1016/j.carpath.2009.12.004. PubMed PMID: 20093049.

8. Guven M, Guven GS, Oz E, Ozaydin A, Batar B, Ulutin T, et al. **DNA repair gene XRCC1 and XPD polymorphisms and their association with coronary artery disease risks and micronucleus frequency**. Heart and vessels. 2007;22(6):355-60. doi: 10.1007/s00380-007-0986-9. PubMed PMID: 18043991.

9. Iarmarcovai G, Bonassi S, Botta A, Baan RA, Orsiere T. **Genetic polymorphisms and micronucleus formation: a review of the literature**. Mutat Res. 2008;658(3):215-33. doi: 10.1016/j.mrrev.2007.10.001. PubMed PMID: 18037339.

10. Rossi ML, Marziliano N, Merlini PA, Ezio Bramucci M, Canosi U, Belli G, et al. **Different Quantitative Apoptotic Traits in Coronary Atherosclerotic Plaques From Patients With Stable Angina Pectoris and Acute Coronary Syndromes**. Circulation. 2004; 110(12):1767-73. doi: 10.1161/01.CIR.0000142865.04816.89. PubMed PMID: 15364800

11. Federici C, Manfredi S, Del Fiandra M, Salpea K, Maubaret C, Humphries SE, et al. **The Common Functional Arg399gln Variant in the DNA Repair Enzyme Xrcc1 Is Associated with Elevated Levels of Micronucleus Chromosomal DNA Damage and Shorter Telomere Length in Patient with Atherosclerosis**. Atherosclerosis. 2009;207(1):e7. doi: 10.1016/j.atherosclerosis.2009.09.047.

12. C. Federici, K.Li KS, Maubaret C, S.E. Humphries, Andreassi MG. **the arg399gln variant in the dna repair enzyme xrcc1 is associated with increased genetic instability in patients with atherosclerosis.** Atherosclerosis Supplements. 2010;11(2):109-22

Second, the following articles were excluded after screening the title and abstract (No.13-17):

13.  Bell R.M, Rossello X, Breckenridge R, Yellon D.M. **Cardioprotective properties of matrix metalloproteinase inhibition:** **Attenuation of nuclear protease activity, reduced DNA fragmentation and preserved OGG1**.European Heart Journal 2015; 36 SUPPL. 1 (170-) (not focus on polymorphism in XRCC1)

14. Skarpengland T, Dahl TB, Skjelland M, Scheffler K, de Sousa MM, Gregersen I, et al. **Enhanced base excision repair capacity in carotid atherosclerosis may protect nuclear DNA but not mitochondrial DNA**. Free Radic Biol Med. 2016; 97:386-97. doi: 10.1016/j.freeradbiomed.2016.07.001. PubMed PMID: 27381496. (the abstract not focus on polymorphisms in XRCC1)

15. Berezina O., Weiner A., Voropaeva E., Pospelova T., Filipenko M. **genetic polymorphism of glutathione s-transferases m1 and t1 (gstm1 and gstt1) in the patients with non-hodgkin malignant.** Haematologica. 2013 98 SUPPL. 1 (631). (couldn’t get the whole article, and the abstract not focus on polymorphisms in XRCC1)

16. Dhillon V S, Thomas P, Iarmarcovai G, et al. **Genetic polymorphisms of genes involved in DNA repair and metabolism influence micronucleus frequencies in human peripheral blood lymphocytes**. Mutagenesis, 2011, 26(1):33-42. (the abstract not focus on Arg194Trp and Arg399Gln in XRCC1);

17. Artwohl M, Lindenmair A, Sexl V, et al. **Different mechanisms of saturated versus polyunsaturated FFA-induced apoptosis in human endothelial cells**. Journal of Lipid Research, 2008, 49(12):2627-40. (the title and abstract not focus on polymorphisms in XRCC1);

The following three studies excluded because of

18. Iarmarcovai G, Bonassi S, Botta A, Baan RA, Orsiere T. **Genetic polymorphisms and micronucleus formation: a review of the literature**. Mutat Res. 2008;658(3):215-33. doi: 10.1016/j.mrrev.2007.10.001. PubMed PMID: 18037339. (this article is review)

19. Federici C, Manfredi S, Del Fiandra M, Salpea K, Maubaret C, Humphries SE, et al. **The Common Functional Arg399gln Variant in the DNA Repair Enzyme Xrcc1 Is Associated with Elevated Levels of Micronucleus Chromosomal DNA Damage and Shorter Telomere Length in Patient with Atherosclerosis**. Atherosclerosis. 2009;207(1):e7. doi: 10.1016/j.atherosclerosis.2009.09.047. (lacking genotype frequency data in the text)

20. Federici C, K.Li KS, Maubaret C, S.E. Humphries, Andreassi MG. **the arg399gln variant in the DNA repair enzyme xrcc1 is associated with increased genetic instability in patients with atherosclerosis**. Atherosclerosis Supplements. 2010;11(2):109-22. (lacking genotype frequency data in the text)

In conclusion, based on the reasons mentioned above, these records are excluded.
